# Long-Term Aortic Remodeling After Thoracic Endovascular Aortic Repair of Acute, Subacute, and Chronic Type B Dissections

**DOI:** 10.3389/fcvm.2022.819501

**Published:** 2022-03-30

**Authors:** Zhenjiang Li, Xiaohui Wang, Yangyan He, Yilang Xiang, Ziheng Wu, Hongkun Zhang, Donglin Li

**Affiliations:** Department of Vascular Surgery, The First Affiliated Hospital, School of Medicine, Zhejiang University, Hangzhou, China

**Keywords:** aorta, dissection, stents, grafting, aortic remodeling

## Abstract

**Objective:**

This study aimed to investigate the characteristics and predictors of aortic remodeling over a long-term follow-up period after thoracic endovascular aortic repair (TEVAR) for acute, subacute, and chronic type B aortic dissections (TBADs).

**Methods:**

Patients who underwent TEVAR for TBAD from July 2011 to December 2013 were included, and relevant data were retrospectively analyzed.

**Results:**

After TEVAR, the true lumen (TL) dimension increased and the false lumen (FL) dimension decreased or did not change over a 5-year follow-up period in all three temporal groups. Shrinkage proportion of the thoracic aorta was the highest in the subacute group (acute, 28.1%; subacute, 39.1%; and chronic, 17.4%; *p* = 0.048), while abdominal expansion showed no significant differences among the groups (acute, 29.6%; subacute, 40.5%; and chronic, 44.4%; *p* = 0.502). The chronic group had a rate of complete FL regression, which is lower than the subacute or acute group at all anatomic sections, with significant differences only in the stented section (chronic, 21.7%; acute, 92.2%; and subacute, 80.4%; *p* < 0.05) and in the distal thoracic aortic section (chronic, 13.0%; acute, 31.1%; and subacute, 50.0%; *p* < 0.05). Logistic regression analysis demonstrated that chronic dissection, TL compression, endoleak, the number of branches from FL, and the number of residual tears affected optimal FL remodeling.

**Conclusion:**

The present study provides data on aortic remodeling of TBAD after TEVAR during a long-term follow-up period. The features and risk factors of aortic remodeling in the acute, subacute, and chronic phases are different in different aortic segments. These findings may have implications in the timing of TEVAR.

## Introduction

Thoracic endovascular aortic repair (TEVAR) is increasingly used in the management of complicated type B aortic dissection (TBAD). Its efficacy has been recognized with a reduction of mortality and morbidity (1). A new challenge is to explore aortic remodeling of the stented aortic segment and the distal aorta postoperatively and during the follow-up period.

Morphological hallmarks of aortic remodeling include false lumen (FL) thrombosis, the shrinkage of FL, and the enlargement of true lumen (TL). FL thrombosis after TEVAR is observed more frequently in acute dissection and the thoracic aorta, particularly in the stented segment. However, for chronic aortic dissection, the reported FL thrombosis rates range from 31 to 91% (2, 3), mainly due to significant heterogeneity in how “chronic” patients are defined. In addition, the morphological changes of the adjacent uncovered thoracic and distal abdominal aortic dissection after TEVAR remain uncertain (4). It has been reported that the absence of FL thrombosis affects favorable aortic remodeling (5), consequently resulting in a higher aorta-related mortality (6), while a higher survival rate can be achieved in patients with adequate aortic remodeling (7). Hence, it is of importance to optimize the strategy and outcome of endovascular therapy for TBAD, identifying the morphological characteristics and influencing factors of aortic remodeling of the dissected thoracic and abdominal aorta.

The aim of this study was to retrospectively examine the mid- and long-term morphological changes of acute, subacute, and chronic TBAD in different segments, including stented thoracic aorta, uncovered thoracic aorta, and abdominal aorta, and to evaluate the factors associated with aortic remodeling.

## Materials and Methods

A retrospective analysis was conducted on consecutive patients who had undergone TEVAR for TBAD from 1 July 2011 to 31 December 2013, at the Department of Vascular Surgery, The First Affiliated Hospital of the Medical School of Zhejiang University. The study was approved by the Institutional Review Board, and informed consent was obtained.

Patients with TBAD were divided into the following three groups according to the interval between symptom onset and TEVAR: acute (≤14 days), subacute (15–90 days), and chronic (> 90 days) groups. Indications for TEVAR were refractory hypertension, persistent or recurrent pain, rupture or impending rupture, malperfusion syndrome, severe compression of TL (TL diameter/aortic diameter <25%), aneurysmal dilatation (maximum thoracic aortic diameter >55 mm), and a rapid growth (≥4 mm within 3 months for both acute and subacute dissection, and ≥4 mm within 1 year for chronic dissection) of the thoracic aorta. Patients with traumatic aortic dissection, underlying inherited diseases (e.g., Marfan syndrome), a residual descending aortic dissection after ascending aorta replacement, and complete FL thrombosis before TEVAR were not included.

### Procedure

Generally, one 15–20-cm-long stent graft with an oversizing rate of about 10% was deployed to cover the major proximal entry tear with proximal landing zones of at least 1.5 cm. The distal landing zones of the stent graft were always above the diaphragm. Adjunctive procedures, including chimney stenting or bypass surgery, were indicated if the proximal landing zones were less than 1.5 cm. Technical success was defined when the stent graft was successfully deployed at the right location, covering the primary tear without proximal endoleak, severe complications, or intraoperative death.

### Follow-up

Follow-up computed tomography angiography (CTA) was routinely performed at 1 month, 6 months, 12 months, and then yearly. The clinical data, including the resolution of pathological conditions and symptoms, complications, re-interventions, and mortality, were recorded at every follow-up.

### Imaging Analysis

Three-dimensional TeraRecon reconstructions were used to obtain the measurements of aortic morphological findings on computed tomography (CT) before the operation (Time 1) and 24 (Time 2), and 60 months (Time 3) after the operation. Patients without postoperative CTA were excluded during an imaging analysis, while patients who had at least one postoperative CTA were still included. For those patients who did not have follow-up CTAs for an imaging analysis at a given time point (24 or 60 months), especially those who died due to aortic events, the measurements of the latest postoperative CTA were used to evaluate their aortic remodeling.

**FIGURE 1:**
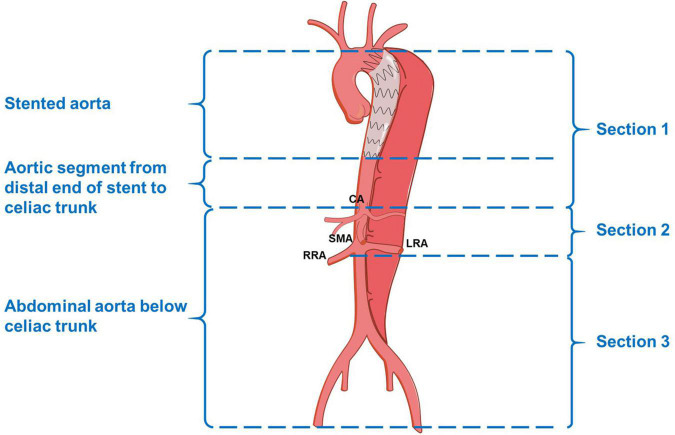
Schematic diagram of the aortic segments and sections. CA, celiac artery; SMA, superior mesenteric artery; RRA, right renal artery; LRA, left renal artery.

**FIGURE 2:**
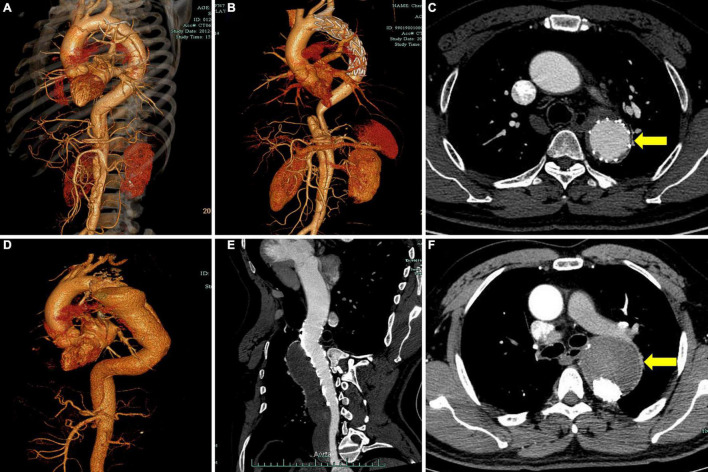
Representative cases of different degrees of FL thrombosis after TEVAR. Computed tomography angiography (CTA) images show the FL status of a patient with subacute aortic dissection before operation **(A)** and 2 years after TEVAR **(B)**. The cross-sectional image shows complete obliteration of FL (**C**, yellow arrow). CTA images show the FL status of a patient with chronic aortic dissection before operation **(D)** and 2 years after TEVAR **(E)**. The cross-sectional image shows total FL thrombosis (**F**, yellow arrow). FL, false lumen; TEVAR, thoracic endovascular aortic repair.

The measurements of the aortic morphological features included maximum thoracic and abdominal aortic diameter, the diameter and cross-sectional area of FL and TL at four levels (the levels of tracheal carina, diaphragm, celiac trunk, and abdominal aorta bifurcation), and the degree of FL thrombosis (based on the latest CTA). We chose the aortic section perpendicular to the regional aorta course and measured the diameters and area manually. The status of the aorta was classified as expansion (maximum aortic diameter increasing by more than 5 mm at a given follow-up time point compared with preoperative measurement), stable (changing by less than 5 mm), and shrinkage (decreasing by more than 5 mm). To clearly describe the distal extension of the dissection and count the number of tears, we divided the target aorta into the following three sections: section 1, thoracic-abdominal aorta above the celiac trunk [i.e., zones 3–5 according to the reporting standards (8)]; section 2, the abdominal aorta between the celiac trunk and renal artery (i.e., zones 6–8); and section 3, the abdominal aorta below the renal artery (i.e., zones 9–11; Figure 1). The degree of FL thrombosis was analyzed at three aortic segments, including stented aorta (stented section 1), an aortic segment between the distal end of the stent and the celiac trunk (non-stented section 1), and the abdominal aorta below the celiac trunk (section 2 + section 3). The status of FL remodeling was classified as complete obliteration (complete thrombosis and total absorption, Figures 2A–C), malabsorption (complete thrombosis with a partial absorption, Figures 2D–F), partial thrombosis, or no thrombosis. Dysremodeling of the FL after TEVAR was defined as any evidence of malabsorption, partial thrombosis, or no thrombosis.

### Statistical Analysis

Statistical analysis was performed using SPSS software (version 19.0; SPSS, Inc., Chicago, IL, United States). Continuous variables were summarized as means ± standard deviations (SDs), and categorical variables were expressed as frequencies. Differences between the three groups were analyzed using the Chi-squared test or Fisher exact test for categorical data and one-way analysis of variance (ANOVA) for continuous variables that satisfied normal distribution. Continuous data without normal distribution were examined using the Kruskal–Wallis test. A repeated measurements ANOVA was applied for analyzing intergroup differences over time. A comparison of postoperative data between the groups was conducted using an analysis of covariance (ANCOVA) or a regression analysis with an adjustment for preoperative data if the data between the groups were significantly different. Univariate and multivariate analyses based on logistic regression were used to assess the factors associated with aortic remodeling. Factors with the value of *p* < 0.05 tested in the univariate analysis were considered for further multivariate analysis. The value of *p* < 0.05 was considered as statistically significant.

## Results

### Patients and Thoracic Endovascular Aortic Repair Outcomes

A total of 141 consecutive patients with TBAD who had received TEVAR during the study period were identified. Among them, 109 (77.3%) were men. The mean age was 54.4 ± 12.4 years (range, 27–84 years). Patients’ preoperative clinical features are presented in Table 1. The technical success rate was 96.5% (136/141) because five patients showed type Ia endoleaks, and these were considered as unsuccessful cases. Considering that the endoleaks were very limited, no additional interventions were conducted. No intraoperative open conversion occurred. A total of 141 stent grafts were implanted, including Valiant (Medtronic, Minneapolis, MN, United States) (*n* = 61), Ankura (Lifetech, Shenzhen, China) (*n* = 54), Zenith TX (Cook Medical, Bloomington, Indiana) (*n* = 17), Hercules (Microport, Shanghai, China) (*n* = 6), and TAG (W.L. Gore & Associates, Flagstaff, AZ, United States) (*n* = 2). The procedural details and clinical outcomes are presented in Table 2. In the early phase, there were no significant differences in major complications (*p* = 0.306), re-intervention (*p* = 0.062), and mortality (*p* = 0.367) among the acute, subacute, and chronic groups. There was no spinal cord ischemia. There were two early deaths, including one due to multiple organ failure (patient from the acute group on postoperative day 29) and another due to an unknown reason (patient from the chronic group on postoperative day 1).

**TABLE 1:** Demographic and preprocedural imaging characteristics of patients with type B aortic dissection (TBAD) receiving an endovascular repair.

	Acute (*N* = 68)	Subacute (*N* = 49)	Chronic (*N* = 24)	*p* *
Age (years, mean ± SD)	55.2 ± 11.9	53.4 ± 12.7	53.7 ± 13.3	0.713
Sex				0.630
Male	53 (77.9%)	36 (73.5%)	20 (83.3%)	
Female	15 (22.1%)	13 (73.5%)	4 (16.7%)	
Hypertension	54 (79.4%)	38 (77.6%)	19 (79.2%)	0.969
Diabetes	2 (2.9%)	2 (4.1%)	1 (4.2%)	0.932
Smoking	33 (48.5%)	20 (40.8%)	12 (50.0%)	0.651
Hyperlipidemia	4 (5.9%)	4 (8.2%)	2 (8.3%)	0.864
Coronary artery disease	3 (4.4%)	3 (6.1%)	1 (4.2%)	0.898
Peripheral artery disease	2 (2.9%)	2 (4.1%)	1 (4.2%)	0.932
Cerebrovascular disease	2 (2.9%)	1 (2.0%)	0 (0%)	0.691
Paralysis	0 (0%)	0 (0%)	0 (0%)	NA
End-stage nephropathy	1 (1.5%)	1 (2.0%)	0 (0%)	0.786
COPD	6 (8.8%)	4 (8.2%)	2 (8.3%)	0.991
Refractory hypertension	14 (20.6%)	9 (18.4%)	0 (0%)	0.057
Intractable pain	15 (22.1%)	10 (20.4%)	0 (0%)	0.043
Visceral malperfusion	21 (30.9%)	20 (40.8%)^‡^	2 (8.3%)^‡^	0.018
Leg ischemia	7 (10.3%)	2 (4.1%)	2 (8.3%)	0.463
True lumen collapse	33 (48.5%)	21 (42.9%)	19 (79.2%)^†^	0.011
Aneurysmal dilation	0 (0%)^†^	5 (10.2%)^†^	16 (70.8%)^†^	0
Rapid enlargement	6 (8.8%)	12 (24.5%)	5 (20.8%)	0.062
Rupture/impending rupture	16 (23.5%)	14 (28.6%)	0 (0%)^†^	0.016
Classification				0.753
DeBakey type IIIa	10 (14.7%)	9 (18.4%)	5 (20.8%)	
DeBakey type IIIb	58 (85.3%)	40 (81.6%)	19 (79.2%)	
Distal extension of dissection				0.316
Section 1	9 (13.2%)	9 (18.4%)	6 (25.0%)	
Section 2	20 (29.4%)	9 (18.4%)	8 (33.3%)	
Section 3	39 (57.4%)	31 (63.3%)	10 (41.7%)	

*p < 0.05 was considered as statistically significant.

*^†^There was a significant difference between the marked group and any one of the other two groups.*

*^‡^There was a significant difference between any two groups with marks. COPD, chronic obstructive pulmonary disease.*

**TABLE 2:** Procedural details and clinical outcomes.

	Acute (*N* = 68)	Subacute (*N* = 49)	Chronic (*N* = 24)	*p* *
Interval (onset to TEVAR, days, mean ± SD)	10.3 ± 4.0	20.5 ± 7.1	365.5 ± 375.7	NA
Procedure time (min, mean ± SD)	103.6 ± 70.5	98.5 ± 70.3	114.1 ± 80.6	0.687
Fluoroscopic time (min, mean ± SD)	14.8 ± 7.6	15.0 ± 9.4	16.9 ± 8.8	0.557
Contrast medium (ml, mean ± SD)	141.2 ± 37.8	129.0 ± 33.6	135.0 ± 41.5	0.216
Length of stay (days, mean ± SD)	16.6 ± 8.0	17.7 ± 6.1	14.9 ± 3.2	0.243
Intraoperative type Ia endoleak	2 (2.9%)	2 (4.1%)	1 (4.2%)	0.932
Technical success	66 (97.1%)	47 (95.9%)	23 (95.8%)	0.932
**Number of stent grafts**				
1	68 (100%)	49 (100%)	24 (100%)	NA
Length of covered aorta (cm, mean ± SD)	15.1 ± 1.5	15.2 ± 1.4	15.6 ± 1.9	0.720
**Stent-graft**				
Diameter (cm, mean ± SD)	3.5 ± 0.3	3.4 ± 0.3	3.5 ± 0.3	0.328
Length (cm, mean ± SD)	16.2 ± 1.5	16.2 ± 1.6	16.7 ± 1.8	0.558
**Adjunctive procedure**				
LSA coverage	11 (16.2%)	11 (22.4%)	7 (29.2%)	0.369
Hybrid operation	1 (1.5%)	1 (2.0%)	1 (4.2%)	0.733
Chimney technique	3 (4.4%)	4 (8.2%)	1 (4.2%)	0.647
**Perioperative outcomes**				
Major complications	10 (14.7%)	3 (6.1%)	2 (8.3%)	0.306
Re-interventions	5 (7.4%)	0 (0%)	0 (0%)	0.062
30-day death	1 (1.5%)	0 (0%)	1 (4.2%)	0.367
Follow-up time (months, mean ± SD)^a^	73.0 ± 34.2	84.5 ± 23.3	74.7 ± 35.0	0.506
** Follow-up outcomes^a^**				
Follow-up major complications	18 (28.1%)	4 (8.7%)^†^	7 (30.4%)	0.028
Follow-up re-interventions	9 (14.1%)	2 (4.3%)	3 (13.0%)	0.238
Follow-up death	7 (10.9%)	3 (6.5%)	3 (13.0%)	0.629

*p < 0.05 was considered as statistically significant.

*^†^There was a significant difference between the marked group and any one of the other two groups.*

*^a^Patients who died or were lost to a follow-up within 30 days were excluded. TEVAR, thoracic endovascular aortic repair; LSA, left subclavian artery; NA, not available.*

The mean follow-up time was 77.2 ± 31.2 months (range: 2–108 months). Eight patients were lost to a follow-up (six patients had no postoperative CTAs due to early missing of the follow-up). All intraoperative type I endoleaks disappeared during a follow-up. One new type Ia endoleak was observed. During the follow-up period, the rate of major complications of the subacute group was significantly lower than that of the acute (8.7 vs. 28.1%, *p* < 0.05) and chronic groups (8.7 vs. 30.4%, *p* < 0.05). There was no report of spinal cord ischemia. The follow-up re-intervention rate (*p* = 0.238) and mortality (*p* = 0.629) among the three groups were not significantly different. The other 13 patients died in the mid- to long-term phase due to aortic rupture (*n* = 8), cerebral hemorrhage (*n* = 1), multiple organ dysfunction syndrome (MODS, *n* = 1), lung infection (*n* = 1), recurrent pericardial effusion and heart failure (*n* = 1), and an unknown reason (*n* = 1).

### Morphological Characteristics of Long-Term Aortic Remodeling

There were eight patients without postoperative CTAs; two of them died postoperatively and six were lost to an early follow-up as mentioned earlier. Hence, during the imaging analysis, 133 patients were included. The anatomical features and follow-up imaging findings are presented in Table 3.

**TABLE 3:** Imaging follow-up results and aortic remodeling.

	Acute (*N* = 64)	Subacute (*N* = 46)	Chronic (*N* = 23)	*p* *
**No. of residual tears**				
Section 1	0.58 ± 0.71	0.39 ± 0.61	0.74 ± 0.86	0.137
Section 2	1.09 ± 0.90	1.22 ± 1.19	1.43 ± 1.31	0.429
Section 3	2.06 ± 1.49	2.02 ± 1.53	2.48 ± 1.16	0.428
No. of branches from FL	1.02 ± 1.03	1.26 ± 1.29	1.09 ± 0.95	0.521
Stent-induced distal erosion	9 (14.1%)^†^	1 (2.2%)	0 (0%)	0.021
Endoleak	6 (9.4%)	4 (8.7%)	2 (8.7%)	0.991
RTAD	4 (6.3%)	0 (0%)	0 (0%)	0.108
Stented thoracic false lumen				<0.001
Complete obliteration	59/64 (92.2%)	37/46 (80.4%)	5/23 (21.7%)^†^	
Dysremodeling	5/64 (7.9%)	9/46 (19.6%)	18/23 (78.3%)	
Malabsorption	4/64 (6.3%)	8/46 (17.4%)	18/23 (78.3%)	
Partial thrombosis	0/64 (0%)	1/46 (2.2%)	0/23 (0%)	
No thrombosis	1/64 (1.6%)	0/46 (0%)	0/23 (0%)	
Distal thoracic false lumen				0.008
Complete obliteration	19/61 (31.1%)	22/44 (50.0%)^‡^	3/23 (13.0%)^‡^	
Dysremodeling	42/61 (68.8%)	22/44 (50.0%)	20/23 (86.9%)	
Malabsorption	11/61 (18.0%)	8/44 (18.2%)	5/23 (21.7%)	
Partial thrombosis	26/61 (42.6%)	12/44 (27.3%)	14/23 (60.9%)	
No thrombosis	5/61 (8.2%)	2/44 (4.5%)	1/23 (4.3%)	
Abdominal false lumen				0.282
Complete obliteration	7/54 (13.0%)	4/37 (10.8%)	0/18 (0%)	
Dysremodeling	47/54 (87.0%)	33/37 (89.1%)	18/18 (100.0%)	
Malabsorption	2/54 (3.7%)	3/37 (8.1%)	1/18 (5.6%)	
Partial thrombosis	26/54 (48.1%)	14/37 (37.8%)	7/18 (38.9%)	
No thrombosis	19/54 (35.2%)	16/37 (43.2%)	10/18 (55.6%)	

*p < 0.05 was considered as statistically significant.

*^†^There was a significant difference between the marked group and any one of the other two groups.*

*^‡^There was a significant difference between any two groups with marks. RTAD, retrograde type A dissection.*

**FIGURE 3:**
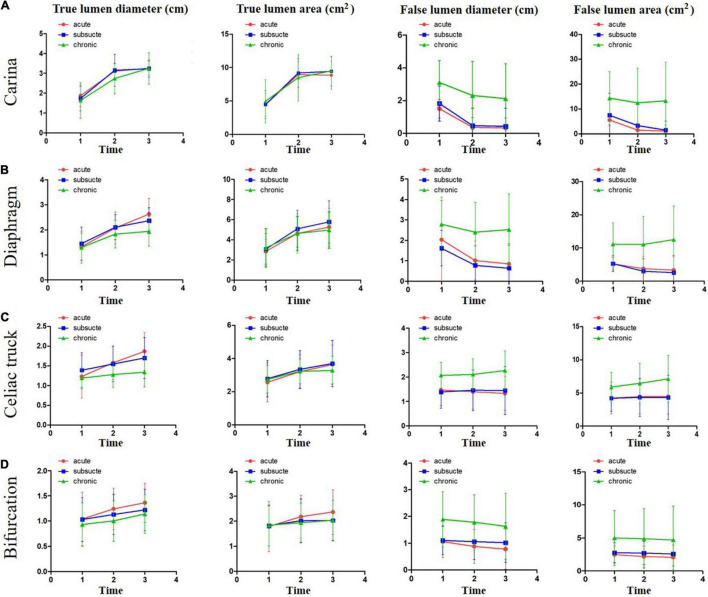
The dimensional changes of TL and FL over a 5-year follow-up period. The changes of TL and FL dimensions over time were plotted for acute, subacute, and chronic aortic dissections after TEVAR at the levels of tracheal carina **(A)**, diaphragm **(B)**, celiac trunk **(C)**, and abdominal aorta bifurcation **(D)**. Time 1, before operation; Time 2, 2 years after operation; and Time 3, 5 years after operation. TL, true lumen; FL, false lumen.

The dimensional changes of TL and FL for all of the groups at all locations are illustrated in Figure 3 and Supplementary Tables 1–4. In general, significant increases in TL diameter and area were documented at all indicated anatomical locations for all of the three groups. The extent of increase in TL diameter and area was greater at the proximal levels than at the distal levels. The change of TL dimension in the acute and subacute groups was greater than that in the chronic group. TL expanded continuously at all levels but slowed down during the late follow-up period (from 24 to 60 months) compared with the early period (from preoperative to 24 months). In contrast, FL remodeling was less favorable. FL diameter and area decreased in the acute and subacute groups at the levels of carina, diaphragm, and abdominal aorta bifurcation and remained unchanged at the level of the celiac trunk over time. The corresponding parameters decreased in the chronic group at the levels of carina and aortic bifurcation but increased at the levels of the diaphragm and the celiac trunk over time. FL expansion was greater and faster during the late follow-up period (from 24 to 60 months) than during the early time (from preoperative to 24 months).

**FIGURE 4:**
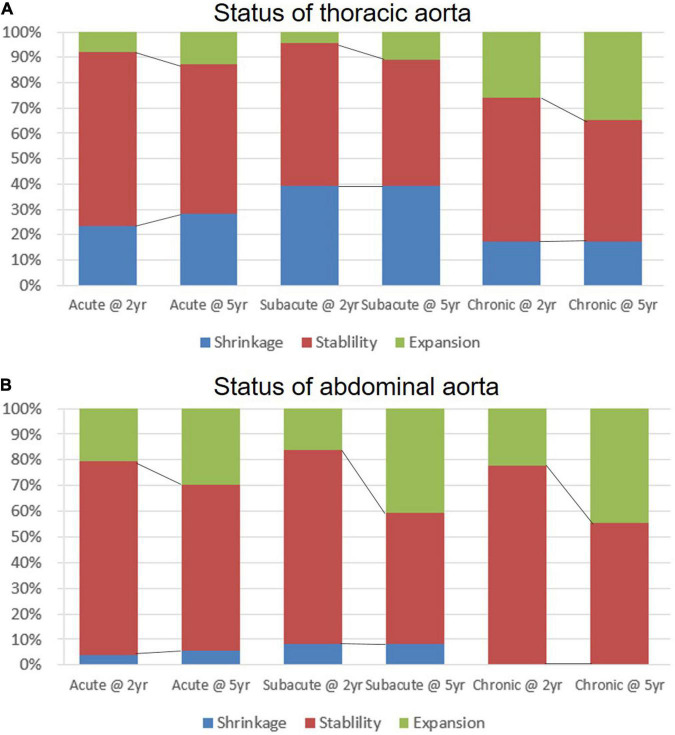
The status of aortic remodeling in the thoracic and abdominal aorta over a 5-year follow-up period. The expansion, stable, or shrinkage status of maximum thoracic **(A)** and abdominal **(B)** aortic diameter observed in acute, subacute, and chronic type B aortic dissections (TBADs) during 5 years of follow-up is shown.

The status of the thoracic and abdominal aorta is illustrated in Figure 4 and Supplementary Table 5. Two years after TEVAR, the thoracic aorta remained stable in more than half of the patients from all of the clinical groups (acute, 68.8%; subacute, 56.5%; and chronic, 56.5%). The proportion of shrinkage appeared to be the highest in the subacute group (acute, 23.4%; subacute, 39.1%; and chronic, 17.4%), while the proportion of expansion was the highest in the chronic group (acute, 7.8%; subacute, 4.4%; and chronic, 26.1%), with a significant difference among the three groups (*p* = 0.016). After 5 years, the proportion of the stable status decreased, and the proportion of expansion increased for all of the groups. The chronic group showed the worst results in terms of aortic remodeling in the thoracic aorta after TEVAR. Over the follow-up period, TEVAR did not significantly reversely modify the abdominal aorta. Patients with unchanged abdominal aorta were still in the majority in all of the groups. In all of the groups, shrinkage occurred in a very low proportion at 2 (acute, 3.7%; subacute, 8.1%; and chronic, 0%) and 5 years (acute, 5.6%; subacute, 8.1%; and chronic, 0%). Expansion occurred more frequently in the abdominal aorta than in the thoracic region, and the frequency increased with time across the three groups. There was no significant difference in abdominal expansion among the three groups at 5 years (*p* = 0.502), despite a tendency toward a higher risk in chronic patients.

**FIGURE 5:**
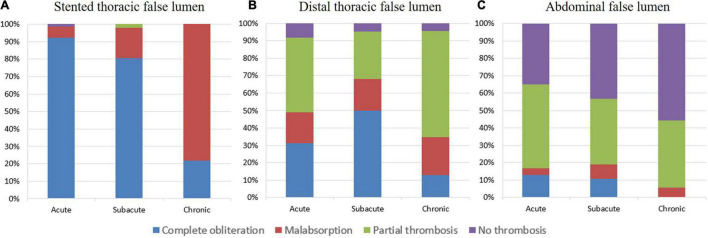
The degree of FL thrombosis in the last follow-up. The complete obliteration, malabsorption, partial thrombosis, or no thrombosis status of FL at the stented thoracic aorta **(A)**, distal thoracic aorta **(B)**, and abdominal aorta **(C)** observed in acute, subacute, and chronic TBADs in the last follow-up.

The degree of FL thrombosis in the three aortic segments at the latest follow-up is shown in Figure 5 and Table 3. The rates of complete FL obliteration were greater as the section was more proximal (the stented thoracic aorta, 75.9%; the thoracic aorta distal to stent, and 34.4%; abdominal aorta, 10.1%) for all of the clinical groups. Chronic patients had a lower rate of complete FL obliteration than subacute or acute patients at all of the sections, but the difference was only significant for the stented section (chronic, 21.7%; acute, 92.2%; and subacute, 80.4%; *p* < 0.05) and the uncovered distal thoracic aortic section (chronic, 13.0%; acute, 31.1%; subacute, 50.0%; and *p* < 0.05).

### Risk Factors of Long-Term Aortic Remodeling

Logistic regression analysis was performed to identify the factors influencing positive FL remodeling in the stented thoracic aorta, non-stented section 1, and abdominal aorta below the celiac trunk (section 2 + section 3) during a long-term follow-up (Table 4). In the multivariate analysis, three factors were associated with the failure of positive aortic remodeling in the stented thoracic aorta, including chronic dissection [odds ratio (OR) 20.0 [3.6–112.0], *p* = 0.001], postoperative endoleak (OR 10.3 [2.4–44.2], *p* = 0.002), and the number of section 2 residual tears (OR 2.2 [1.3–3.8], *p* = 0.004). Four factors influenced positive FL remodeling in non-stented section 1, including TL compression (OR 2.9 [1.1–7.9], *p* = 0.033), the number of visceral branches from FL (OR 1.9 [1.1–3.3], *p* = 0.003), the number of section 1 residual tears (OR 6.9 [2.6–18.8], *p* < 0.001), and number of section 2 residual tears (OR 1.9 [1.0–3.6], *p* = 0.046). Four factors were associated with optimal FL remodeling failure in the abdominal aorta below the celiac trunk, including TL compression (OR 25.7 [1.2–567.1], *p* = 0.040), the number of visceral branches from FL (OR 34.0 [1.1–1,089.8], *p* = 0.046), the number of section 2 residual tears (OR 17.9 [1.3–240.5], *p* = 0.030), and the number of section 3 residual tears (OR 3.0 [1.1–8.6], *p* = 0.039).

**TABLE 4:** Analysis of risk factors influencing aortic remodeling of false lumen.

	Stented thoracic aortic FL		Distal thoracic aortic FL		Abdominal aortic FL	
	Multivariate analysis [OR (95% CI)]^†^	*p*	Multivariate analysis [OR (95% CI)]^†^	*p*	Multivariate analysis [OR (95% CI)]^†^	*p*
True lumen collapse			2.9 (1.1–7.9)	0.033	25.7 (1.2–567.1)	0.040
Aneurysmal dilation	3.2 (0.5–21.7)	0.236				
Chronic dissection (vs. non-chronic dissection)	20.0 (3.6–112.0)	0.001	4.3 (0.9–20.2)	0.068		
No. of branches from FL			1.9 (1.1–3.3)	0.033	34.0 (1.1–1089.8)	0.046
No. of residual tears in section 1			6.9 (2.6–18.8)	0		
No. of residual tears in section 2	2.2 (1.3–3.8)	0.004	1.9 (1.0–3.6)	0.046	17.9 (1.3–240.5)	0.030
No. of residual tears in section 3			1.0 (0.7–1.4)	0.954	3.0 (1.1–8.6)	0.039
Endoleak	10.3 (2.4–44.2)	0.002				

*OR, odds ratio; CI, confidence interval; FL, false lumen.*

*^†^Factors tested in the univariate analysis with p < 0.05 were considered for further multivariate analysis. Data are presented as the OR (95% CI) and p-value. The value of p < 0.05 was considered as statistically significant.*

## Discussion

Because it is now accepted to divide the course of an aortic dissection into acute, subacute, and chronic phases (1), it is of clinical relevance to determine aortic remodeling at the different phases of TBAD. However, the available data are scarce and are based on small sample sizes and short follow-up periods. This single-center, retrospective, observational study provided more data on aortic remodeling in different temporal groups during a long-term follow-up period. Aortic remodeling was shown to persist even in the late phase after TEVAR. The degree of aortic remodeling in patients with subacute TBAD was similar to that in patients with acute TBAD and greater than that in patients with chronic TBAD. Remodeling in stented aortic segments was greater than that in uncovered distal thoracic aorta and abdominal aorta. Chronic dissection, endoleak, preoperative TL compression, the number of residual tears, and the number of branches from FL were identified as risk factors in a long-term follow-up.

Several studies have provided valuable data related to aortic remodeling of acute, subacute, and chronic dissections. According to Zhou et al., during a 6-month follow-up period, there was no significant difference in aortic remodeling in terms of diameters of TL and FL and TL index between acute and subacute patients (9). The VIRTUE Registry Investigation revealed that during a 3-year follow-up period, the subacute group had significantly greater FL area regression than the chronic group, while there was no significant difference between subacute and acute patients (10). Our results demonstrated that during a 5-year follow-up period, aortic remodeling in the late phase maintained the trend, while FL remodeling was the worst in the chronic group. These findings suggest that aortic plasticity is still high enough in the subacute phase, and favorable aortic remodeling can be expected.

False lumen thrombosis has been linked to clinical success and has been associated with long-term outcomes (11). The VIRTUE study revealed that the FL thrombosis rate was lower in chronic dissections than in acute and subacute dissections in all anatomical regions (10). The present study confirmed these results in a long-term period, i.e., the rate of FL thrombosis was lower in chronic dissections and in more distal aorta. Assisted FL management after stent graft deployment in TL could be considered to promote FL thrombosis and improve clinical outcomes, and several FL intervention techniques have been proposed with favorable outcomes.

Previous studies have revealed an unstable abdominal aorta after TEVAR. Andacheh et al. reported the expansion of infrarenal aortic FL diameter and overall abdominal aortic volume after TEVAR (12). Steingruber et al. also observed aortic expansion from the distal end of the stent graft to the celiac artery (13). In this study, abdominal aortic expansion was observed in all three groups, and the proportion increased with time during a long-term follow-up, indicating the necessity of paying more attention to abdominal FL and providing appropriate interventions on time.

In this study, chronic dissection, endoleak, and residual tears in section 2 were identified as predictive of incomplete FL regression in the stented aorta. With regard to the natural transition of an aortic dissection from acute to chronic stages, the intimal flap dynamically changes over time *via* thickening, straightening, and the loss of mobility. This significant change may decrease the possibility of the dissection flap to be re-approximated to the aortic wall after stent graft placement, thereby making the elimination of FL and aortic remodeling more difficult (14). Hence, the most recent guidelines from the Society of Thoracic Surgeons and the American Association for Thoracic Surgery (STS/AATS) recommend considering open surgical repair for patients with chronic dissection unless comorbidities are prohibitive, while TEVAR can be considered for patients with a high surgical risk and suitable anatomy (15). However, high-quality evidence from clinical trials that compare open surgery and TEVAR for chronic TBAD is very limited. Obviously, endoleak would permit persistent FL blood flow and continuous FL pressurization, leading to remodeling failure. A moderate-to-severe endoleak with a large flow found intraoperatively or at a follow-up should be managed with endograft extension or embolization.

Our study showed that TL compression, the number of branches originating from FL, and the number of residual tears were the risk factors affecting complete FL regression in the distal dissected aorta. Previous studies have indicated branches arising from FL and the distal residual tear as the risk factors related to failed FL regression (16, 17). A plausible explanation would be that multiple uncovered distal tears after TEVAR, which are common in DeBakey IIIb aortic dissections, provide adequate inflow for abdominal and thoracic FL, and FL branches function as an outflow tract for the persistent retrograde blood flow from distal tears in FL, which may result in continuous FL pressurization and the failure of complete thrombosis. Based on this possible pathophysiologic mechanism, patients with an increased risk of aortic dilatation should be treated more aggressively with extended endograft coverage to the celiac artery or fenestrated/branched stent grafting (18) to cover more distal entry tears; however, the risk of spinal cord ischemia should be balanced. Preemptive embolization of FL and its branches is a newly proposed approach worth trying to improve FL regression (17) with an occluder, a plug, glue, and coils (19, 20).

Narrow TL is related to higher FL pressure and greater severity and complexity of the lesion. As reported in previous studies, the decreased preoperative abdominal aorta TL area ratio is associated with patent FL (16). Hence, an endovascular approach to expand the TL of distal aorta following the proximal TEVAR procedure may be necessary to promote favorable aortic remodeling. Recently, several novel devices and techniques have been proposed to achieve better aortic remodeling, such as composite stent (21), PETTICOAT (22), and STABILISE (23), technique to prolong the stent coverage length and increase the TL dimension. Aortic septotomy with a laser or cheese wire is another valuable strategy to create common aortic lumen, optimize the landing zone, and avoid persistent distal FL perfusion, which can remarkably improve aortic remodeling and survival of chronic dissection patients (24). Although more robust clinical data are needed, these approaches seem to be promising.

## Limitations

This was a retrospective observational study with a relatively small number of patients, which limited the statistical power. In addition, a wide range of different types of stent grafts were used, which may also be a source of heterogeneity.

## Conclusion

Favorable aortic remodeling can be achieved after a successful TEVAR for TBAD, which could be affected by the phase of the dissection and the aortic segments. The subacute phase may provide a potentially attractive therapeutic window to perform TEVAR. Postoperative surveillance is important to detect the adverse progression of unstented aortic segments. Chronic dissection, TL compression, endoleak, the number of branches from FL, and the number of residual tears are the risk factors that affect optimal FL remodeling. More patients and a longer follow-up period are needed to make robust conclusions.

## Data Availability Statement

The raw data supporting the conclusions of this article will be made available by the authors, without undue reservation.

## Ethics Statement

The studies involving human participants were reviewed and approved by the Ethics Committee of the First Affiliated Hospital of the Medical School of Zhejiang University. The patients/participants provided their written informed consent to participate in this study.

## Author Contributions

HZ and DL were involved in the concept and design. ZL, XW, YH, and YX were involved in the analysis and interpretation of data. ZL and XW were involved in writing the manuscript. ZL, XW, HZ, and DL were involved in the critical revision of this manuscript. ZW were involved in statistical analysis. ZL, HZ, and DL were involved in fund raising. All authors contributed to the article and approved the submitted version.

## Conflict of Interest

The authors declare that the research was conducted in the absence of any commercial or financial relationships that could be construed as a potential conflict of interest.

## Publisher’s Note

All claims expressed in this article are solely those of the authors and do not necessarily represent those of their affiliated organizations, or those of the publisher, the editors and the reviewers. Any product that may be evaluated in this article, or claim that may be made by its manufacturer, is not guaranteed or endorsed by the publisher.

## Abbreviations

TEVAR: thoracic endovascular aortic repair
TBAD: type B aortic dissection
FL: false lumen
TL: true lumen
CTA: computed tomography angiography.

## References

[1] Erbel R Aboyans V Boileau C Bossone E Bartolomeo RD Eggebrecht H 2014 ESC guidelines on the diagnosis and treatment of aortic diseases: document covering acute and chronic aortic diseases of the thoracic and abdominal aorta of the adult. The task force for the diagnosis and treatment of aortic diseases of the European society of cardiology (ESC). *Eur Heart J.* (2014) 35:2873–926. 10.1093/eurheartj/ehu281 25173340

[2] Nienaber CA Rousseau H Eggebrecht H Kische S Fattori R Rehders TC Randomized comparison of strategies for type B aortic dissection: the INvestigation of STEnt grafts in aortic dissection (INSTEAD) trial. *Circulation.* (2009) 120:2519–28. 10.1161/CIRCULATIONAHA.109.886408 19996018

[3] Kang WC Greenberg RK Mastracci TM Eagleton MJ Hernandez AV Pujara AC Endovascular repair of complicated chronic distal aortic dissections: intermediate outcomes and complications. *J Thorac Cardiovasc Surg.* (2011) 142:1074–83. 10.1016/j.jtcvs.2011.03.008 21549398

[4] Nienaber CA Kische S Rousseau H Eggebrecht H Rehders TC Kundt G Endovascular repair of type B aortic dissection: long-term results of the randomized investigation of stent grafts in aortic dissection trial. *Circ Cardiovasc Interv.* (2013) 6:407–16. 10.1161/CIRCINTERVENTIONS.113.000463 23922146

[5] Nienaber CA Kische S Akin I Rousseau H Eggebrecht H Fattori R Strategies for subacute/chronic type B aortic dissection: the Investigation of stent grafts in patients with type B aortic dissection (INSTEAD) trial 1-year outcome. *J Thorac Cardiovasc Surg.* (2010) 140:S101–8. 10.1016/j.jtcvs.2010.07.026 21092774

[6] Mani K Clough RE Lyons OT Bell RE Carrell TW Zayed HA Predictors of outcome after endovascular repair for chronic type B dissection. *Eur J Vasc Endovasc Surg.* (2012) 43:386–91. 10.1016/j.ejvs.2012.01.016 22326695

[7] Watanabe Y Shimamura K Yoshida T Daimon T Shirakawa Y Torikai K Aortic remodeling as a prognostic factor for late aortic events after thoracic endovascular aortic repair in type B aortic dissection with patent false lumen. *J Endovasc Ther.* (2014) 21:517–25. 10.1583/13-4646R.1 25101579

[8] Fillinger MF Greenberg RK McKinsey JF Chaikof EL. Society for vascular surgery Ad Hoc committee on TEVAR reporting standards. reporting standards for thoracic endovascular aortic repair (TEVAR). *J Vasc Surg.* (2010) 52:1022–33. 10.1016/j.jvs.2010.07.008 20888533

[9] Zhou W Yu W Wang Y Li Y Sheng W Wang Q Assessing aortic remodeling after thoracic endovascular aortic repair (TEVAR) in DeBakey IIIb aortic dissection: a retrospective study. *Ann Thorac Cardiovasc Surg.* (2019) 25:46–55. 10.5761/atcs.oa.18-00167 30305479 PMC6388301

[10] Virtue Registry Investigators. Mid-term outcomes and aortic remodelling after thoracic endovascular repair for acute, subacute, and chronic aortic dissection: the VIRTUE Registry. *Eur J Vasc Endovasc Surg.* (2014) 48:363–71. 10.1016/j.ejvs.2014.05.007 24952999

[11] Stanley GA Murphy EH Knowles M Ilves M Jessen ME Dimaio JM Volumetric analysis of type B aortic dissections treated with thoracic endovascular aortic repair. *J Vasc Surg.* (2011) 54:985–92. 10.1016/j.jvs.2011.03.263 21917398

[12] Andacheh ID Donayre C Othman F Walot I Kopchok G White R. Patient outcomes and thoracic aortic volume and morphologic changes following thoracic endovascular aortic repair in patients with complicated chronic type B aortic dissection. *J Vasc Surg.* (2012) 56:644–50. 10.1016/j.jvs.2012.02.050 22640467

[13] Steingruber IE Chemelli A Glodny B Hugl B Bonatti J Hiemetzbeger R Endovascular repair of acute type B aortic dissection: midterm results. *J Endovasc Ther.* (2008) 15:150–60. 18426272 10.1583/07-2288.1

[14] Lee M Lee do Y Kim MD Lee MS Won JY Park SI Outcomes of endovascular management for complicated chronic type B aortic dissection: effect of the extent of stent graft coverage and anatomic properties of aortic dissection. *J Vasc Interv Radiol.* (2013) 24:1451–60. 10.1016/j.jvir.2013.06.007 23932416

[15] MacGillivray TE Gleason TG Patel HJ Aldea GS Bavaria JE Beaver TM The society of thoracic surgeons/American association for thoracic surgery clinical practice guidelines on the management of type B aortic dissection. *J Thorac Cardiovasc Surg.* (2022). [Epub ahead of print] 10.1016/j.jtcvs.2021.11.091 35090765

[16] Chen IM Chen PL Huang CY Weng SH Chen WY Shih CC. Factors affecting optimal aortic remodeling after thoracic endovascular aortic repair of type B (IIIb) aortic dissection. *Cardiovasc Interv Radiol.* (2017) 40:671–81. 10.1007/s00270-017-1563-y 28116473

[17] Liu F Ge YY Guo W Liu XP Jia X Xiong J Preoperative thoracic false lumen branches are predictors of aortic enlargement after stent grafting for DeBakey IIIb aortic dissection. *J Thorac Cardiovasc Surg.* (2018) 155:21–9.e3. 10.1016/j.jtcvs.2017.09.010 29017791

[18] Oikonomou K Kasprzak P Katsargyris A Marques De Marino P Pfister K Verhoeven ELG Mid-term results of fenestrated/branched stent grafting to treat post-dissection thoraco-abdominal aneurysms. *Eur J Vasc Endovasc Surg.* (2019) 57:102–9. 10.1016/j.ejvs.2018.07.032 30181064

[19] Yuan X Mitsis A Semple T Castro Verdes M Cambronero-Cortinas E Tang Y False lumen intervention to promote remodelling and thrombosis-the FLIRT concept in aortic dissection. *Catheter Cardiovasc Interv.* (2018) 92:732–40. 10.1002/ccd.27599 29602262

[20] Pellenc Q Roussel A De Blic R Girault A Cerceau P Ben Abdallah I False lumen embolization in chronic aortic dissection promotes thoracic aortic remodeling at midterm follow-up. *J Vasc Surg.* (2019) 70:710–7. 10.1016/j.jvs.2018.11.038 30850289

[21] Sobocinski J Lombardi JV Dias NV Berger L Zhou Q Jia F Volume analysis of true and false lumens in acute complicated type B aortic dissections after thoracic endovascular aortic repair with stent grafts alone or with a composite device design. *J Vasc Surg.* (2016) 63:1216–24. 10.1016/j.jvs.2015.11.037 26806523

[22] Bertoglio L Rinaldi E Melissano G Chiesa R. The PETTICOAT concept for endovascular treatment of type B aortic dissection. *J Cardiovasc Surg (Torino).* (2019) 60:91–9. 10.23736/S0021-9509.17.09744-0 28183174

[23] Faure EM El Batti S Abou Rjeili M Julia P Alsac JM. Mid-term outcomes of stent assisted balloon induced intimal disruption and relamination in aortic dissection repair (STABILISE) in acute type B aortic dissection. *Eur J Vasc Endovasc Surg.* (2018) 56:209–15. 10.1016/j.ejvs.2018.04.008 29891434

[24] Fukuhara S Khaja MS Williams DM Marko X Yang B Patel HJ Aortic septotomy to optimize landing zones during thoracic endovascular aortic repair for chronic type B aortic dissection. *J Thorac Cardiovasc Surg.* (2021). [Epub ahead of print] 10.1016/j.jtcvs.2021.07.049 34509296

