# Supplementary material for: Long-Term Aortic Remodeling After Thoracic Endovascular Aortic Repair of Acute, Subacute, and Chronic Type B Dissections
Source: Front Cardiovasc Med. 2022 Mar 30;9:819501. doi: 10.3389/fcvm.2022.819501 (PMC9005851; doi:10.3389/fcvm.2022.819501)
Supplement: Supplementary file 1 [file Data_Sheet_1.docx]

Supplementary Table 1 Comparison of the true lumen diameters before and after TEVAR among the acute, subacute and chronic groups

| Level/group | TL diameter (cm) | | | p value for difference for over time among groups |
| --- | --- | --- | --- | --- |
|  | Preoperative | 24 months FU | 60 months FU |  |
| **Tracheal carina level** |  |  |  | 0.165 |
| Acute (n = 64) | 1.87 ± 0.55 | 3.11 ± 0.37^§^ | 3.25 ± 0.34^§¶^ |  |
| Subacute (n = 46) | 1.73 ± 0.62 | 3.15 ± 0.81^§^ | 3.23 ± 0.42^§^ |  |
| Chronic (n = 23) | 1.78 ± 0.65 | 2.74 ± 0.77^§^ | 3.25 ± 0.79^§¶^ |  |
| p value among groups | 0.269 | 0.067 | 0.818 |  |
| **Diaphragm level** |  |  |  | <0.001^‡^ |
| Acute (n=59) | 1.32 ± 0.55 | 2.08 ± 0.63^§^ | 2.63 ± 0.63^§¶^ |  |
| Subacute (n=45) | 1.45 ± 0.66 | 2.11 ± 0.50^§^ | 2.37 ± 0.52 ^§¶^ |  |
| Chronic (n=23) | 1.30 ± 0.63 | 1.83 ± 0.55^§^ | 1.94 ± 0.59^§¶^ |  |
| p value among groups | 0.481 | 0.127 | <0.001^*^ |  |
| **Celiac trunk level** |  |  |  | 0.026^‡^ |
| Acute (n=54) | 1.23 ± 0.54 | 1.58 ± 0.42^§^ | 1.87 ± 0.48^§¶^ |  |
| Subacute (n=37) | 1.39 ± 0.44 | 1.55 ± 0.45^§^ | 1.70 ± 0.52^§¶^ |  |
| Chronic (n=18) | 1.19 ± 0.27 | 1.28 ± 0.33 | 1.34 ± 0.37^§¶^ |  |
| p value among groups | 0.265 | 0.027^*^ | <0.001^*^ |  |
| **Abdominal aortic bifurcation level** |  |  |  | 0.362 |
| Acute (n=40) | 1.04 ± 0.53 | 1.24 ± 0.41^§^ | 1.36 ± 0.39^§¶^ |  |
| Subacute (n=29) | 1.03 ± 0.43 | 1.13 ± 0.40 | 1.22 ± 0.41^§¶^ |  |
| Chronic (n=10) | 0.93 ± 0.43 | 1.00 ± 0.40 | 1.13 ± 0.38^§¶^ |  |
| p value among groups | 0.813 | 0.211 | 0.177 |  |

Data presented as mean ± standard deviation.

^§^ p<0.05, significant difference within groups as comparing with preoperative data.

^¶^ p<0.05, significant difference within groups as comparing with postoperative 24 months data.

^*^ p<0.05, significant difference among groups at a given follow-up time point.

^‡^ p<0.05, significant difference among groups over time.

FU, follow up

Supplementary Table 2 Comparison of the true lumen areas before and after TEVAR among the acute, subacute and chronic groups

| Level/group | TL area (cm^2^) | | | p value for difference for over time among groups |
| --- | --- | --- | --- | --- |
|  | Preoperative | 24 months FU | 60 months FU |  |
| **Tracheal carina level** |  |  |  | 0.682 |
| Acute (n=64) | 4.43 ± 1.73 | 8.96 ± 1.92^§^ | 8.86 ± 2.09^§^ |  |
| Subacute (n=46) | 4.47 ± 2.06 | 9.19 ± 2.13^§^ | 9.44 ± 2.18^§^ |  |
| Chronic (n=23) | 4.96 ± 3.23 | 8.49 ± 3.50^§^ | 9.50 ± 2.22^§¶^ |  |
| p value among groups | 0.890 | 0.387 | 0.275 |  |
| **Diaphragm level** |  |  |  | 0.402 |
| Acute (n=59) | 2.85 ± 1.18 | 4.62 ± 1.95^§^ | 5.25 ± 1.88^§¶^ |  |
| Subacute (n=45) | 3.07 ± 1.57 | 5.09 ± 1.84^§^ | 5.77 ± 2.10^§¶^ |  |
| Chronic (n=23) | 3.20 ± 1.90 | 4.62 ± 1.71^§^ | 4.96 ± 1.80^§¶^ |  |
| p value among groups | 0.959 | 0.410 | 0.216 |  |
| **Celiac trunk level** |  |  |  | 0.746 |
| Acute (n=54) | 2.57 ± 1.18 | 3.21 ± 1.03^§^ | 3.64 ± 1.16^§¶^ |  |
| Subacute (n=37) | 2.79 ± 1.09 | 3.35 ± 1.14^§^ | 3.70 ± 1.39^§¶^ |  |
| Chronic (n=18) | 2.75 ± 0.83 | 3.23 ± 0.74^§^ | 3.29 ± 0.85^§^ |  |
| p value among groups | 0.600 | 0.815 | 0.466 |  |
| **Abdominal aortic bifurcation level** |  |  |  | 0.679 |
| Acute (n=40) | 1.79 ± 1.00 | 2.18 ± 0.88^§^ | 2.37 ± 0.89^§¶^ |  |
| Subacute (n=29) | 1.82 ± 0.80 | 2.02 ± 0.87 | 2.03 ± 0.81 |  |
| Chronic (n=10) | 1.84 0.82 | 1.94 ± 0.77^§^ | 2.04 ± 0.80 |  |
| p value among groups | 0.985 | 0.620 | 0.204 |  |

Data presented as mean ± standard deviation.

^§^ p<0.05, significant difference within groups as comparing with preoperative data.

^¶^ p<0.05, significant difference within groups as comparing with postoperative 24 months data.

FU, follow up

Supplementary Table 3 Comparison of the preoperative false lumen diameters with that at postoperative follow-up times among the acute, subacute and chronic groups

| Level/group | FL diameter (cm) | | | p value for difference for over time among groups |
| --- | --- | --- | --- | --- |
|  | Preoperative | 24 months FU | 60 months FU |  |
| **Tracheal carina level** |  |  |  | <0.001^‡^ |
| Acute (n=64) | 1.51 ± 0.57 | 0.37 ± 0.59^§^ | 0.33 ± 0.60^§^ |  |
| Subacute (n=46) | 1.82 ± 1.07 | 0.47 ± 1.06^§^ | 0.43 ± 1.10^¶^ |  |
| Chronic (n=23) | 3.11 ± 1.33 | 2.31 ± 2.08^§^ | 2.12 ± 2.14^§¶^ |  |
| p value among groups | <0.001^*^ | <0.001^*^ | <0.001^*^ |  |
| **Diaphragm level** |  |  |  | <0.001^‡^ |
| Acute (n=59) | 2.04 ± 2.08 | 1.02 ± 0.96^§^ | 0.85 ± 0.99^§¶^ |  |
| Subacute (n=45) | 1.62 ± 0.87 | 0.78 ± 0.95^§^ | 0.64 ± 1.13^§^ |  |
| Chronic (n=23) | 2.79 ± 1.16 | 2.40 ± 1.45^§^ | 2.53 ± 1.75 |  |
| p value among groups | 0.019^*^ | <0.001^*^ | <0.001^*^ |  |
| **Celiac trunk level** |  |  |  | <0.001^‡^ |
| Acute (n=54) | 1.47 ± 0.63 | 1.40 ± 0.70 | 1.33 ± 0.69 |  |
| Subacute (n=37) | 1.39 ± 0.66 | 1.46 ± 0.83 | 1.45 ± 0.98 |  |
| Chronic (n=18) | 2.07 ± 0.53 | 2.11 ± 0.64 | 2.27 ± 0.80^§¶^ |  |
| p value among groups | 0.001^*^ | 0.002^*^ | 0.001^*^ |  |
| **Abdominal aortic bifurcation level** |  |  |  | 0.003^‡^ |
| Acute (n=40) | 1.06 ± 0.59 | 0.88 ± 0.64^§^ | 0.78 ± 0.75^§¶^ |  |
| Subacute (n=29) | 1.10 ± 0.53 | 1.06 ± 0.67 | 1.02 ± 0.74 |  |
| Chronic (n=10) | 1.89 ± 1.03 | 1.78 ± 1.03^§^ | 1.63 ± 1.25^§^ |  |
| p value among groups | 0.002^*^ | 0.003^*^ | 0.015^*^ |  |

Data presented as mean ± standard deviation.

^§^ p<0.05, significant difference within groups as comparing with preoperative data.

^¶^ p<0.05, significant difference within groups as comparing with postoperative 24 months data.

^*^ p<0.05, significant difference among groups at a given follow-up time point.

^‡^ p<0.05, significant difference among groups over time.

FU, follow up

Supplementary Table 4 Comparison of the preoperative false lumen areas with that at postoperative follow-up times among the acute, subacute and chronic groups

| Level/group | FL area (cm^2^) | | | p value for difference for over time among groups |
| --- | --- | --- | --- | --- |
|  | Preoperative | 24 months FU | 60 months FU |  |
| **Tracheal carina level** |  |  |  | <0.001^‡^ |
| Acute (n=64) | 5.62 ± 2.31 | 1.55 ± 2.62^§^ | 1.14 ± 2.42^§^ |  |
| Subacute (n=46) | 7.50 ± 8.81 | 3.35 ± 8.41^§^ | 1.55 ± 3.60^§^ |  |
| Chronic (n=23) | 14.38 ± 10.62 | 12.53 ± 13.88 | 13.29 ± 15.47 |  |
| p value among groups | <0.001^*^ | <0.001^*^ | <0.001^*^ |  |
| **Diaphragm level** |  |  |  | <0.001^‡^ |
| Acute (n=59) | 5.24 ± 1.90 | 3.76 ± 3.75^§^ | 3.37 ± 4.02^§¶^ |  |
| Subacute (n=45) | 5.28 ± 2.34 | 3.01 ± 3.70^§^ | 2.58 ± 5.23^§^ |  |
| Chronic (n=23) | 11.13 ± 6.44 | 11.08 ± 8.41 | 12.53 ± 10.13^¶^ |  |
| p value among groups | <0.001^*^ | <0.001^*^ | <0.001^*^ |  |
| **Celiac trunk level** |  |  |  | 0.006^‡^ |
| Acute (n=54) | 4.22 ± 2.40 | 4.45 ± 2.60 | 4.45 ± 2.63 |  |
| Subacute (n=37) | 4.19 ± 1.95 | 4.34 ± 2.86 | 4.30 ± 3.34 |  |
| Chronic (n=18) | 5.88 ± 2.22 | 6.47 ± 3.01 | 7.12 ± 3.53^§¶^ |  |
| p value among groups | 0.017^*^ | 0.017^*^ | 0.003^*^ |  |
| **Abdominal aortic bifurcation level** |  |  |  | 0.003^‡^ |
| Acute (n=40) | 2.47 ± 1.31 | 2.19 ± 1.62^§^ | 2.08 ± 1.85 |  |
| Subacute (n=29) | 2.78 ± 1.50 | 2.72 ± 1.76 | 2.60 ± 1.84 |  |
| Chronic (n=10) | 5.00 ± 4.15 | 4.89 ± 4.52 | 4.72 ± 5.10 |  |
| p value among groups | 0.035^*^ | 0.037^*^ | 0.067 |  |

Data presented as mean ± standard deviation.

^§^ p<0.05, significant difference within groups as comparing with preoperative data.

^¶^ p<0.05, significant difference within groups as comparing with postoperative 24 months data.

^*^ p<0.05, significant difference among groups at a given follow-up time point.

^‡^ p<0.05, significant difference among groups over time.

FU, follow up

Supplementary Table 5 Changes of maximum aortic diameters of thoracic and abdominal aorta in acute, subacute and chronic type B aortic dissections at 2 years and 5 years follow up.

|  | Acute @2yr FU  (N = 64) | Subacute @2yr FU  (N = 46) | Chronic @2yr FU  (N = 23) | P^*^ | Acute @5yr FU  (N = 64) | Subacute @5yr FU  (N = 46) | Chronic @5yr FU  (N =23) | P^*^ |
| --- | --- | --- | --- | --- | --- | --- | --- | --- |
| Φ_max_ of thoracic aorta |  |  |  | ***0.016*** |  |  |  | ***0.048*** |
| Shrinkage | 15(23.4%) | 18(39.13%) | 4(17.4%) |  | 18(28.1%) | 18(39.1%) | 4(17.4%) |  |
| Stability | 44(68.8%) | 26(56.52%) | 13(56.5%) |  | 38(59.4%) | 23(50%) | 11(47.8%) |  |
| Expansion | 5(7.8%) | 2(4.35%) | 16(26.1%) |  | 8(12.5%)) | 5(10.9%) | 8(34.8%) |  |
|  | Acute @2yr FU  (N = 54) | Subacute @2yr FU  (N = 37) | Chronic @2yr FU  (N = 18) | P | Acute @5yr FU  (N = 54) | Subacute @5yr FU  (N = 37) | Chronic @5yr FU  (N = 18) | P |
| Φ_max_ of abdominal aorta |  |  |  | 0.695 |  |  |  | 0.502 |
| Shrinkage | 2(3.7%) | 3(8.1%) | 0(0%) |  | 3(5.6%) | 3(8.1%) | 0(0%) |  |
| Stability | 41(75.9%) | 28(75.7%) | 14(77.8%) |  | 35(64.8%) | 19(51.4%) | 10(55.6%) |  |
| Expansion | 11(20.4%) | 6(16.2%) | 4(22.2%) |  | 16(29.6%) | 15(40.5%) | 8(44.4%) |  |

Expansion means maximum aortic diameter increasing greater than 5 mm at a given follow-up time point compared with preoperative measurement; Stability means changing less than 5 mm; and Shrinkage means decreasing greater than 5 mm.

^*^ A P value < 0.05 was considered statistically significant.

@, at; yr, years; FU, follow up;
